# Machine learning prediction of atrial fibrillation in cardiovascular patients using cardiac magnetic resonance and electronic health information

**DOI:** 10.3389/fcvm.2022.998558

**Published:** 2022-09-28

**Authors:** Steven Dykstra, Alessandro Satriano, Aidan K. Cornhill, Lucy Y. Lei, Dina Labib, Yoko Mikami, Jacqueline Flewitt, Sandra Rivest, Rosa Sandonato, Patricia Feuchter, Andrew G. Howarth, Carmen P. Lydell, Nowell M. Fine, Derek V. Exner, Carlos A. Morillo, Stephen B. Wilton, Marina L. Gavrilova, James A. White

**Affiliations:** ^1^Stephenson Cardiac Imaging Centre, Libin Cardiovascular Institute of Alberta, University of Calgary, Calgary, AB, Canada; ^2^Department of Cardiac Sciences, Cumming School of Medicine, University of Calgary, Calgary, AB, Canada; ^3^Department of Diagnostic Imaging, Cumming School of Medicine, University of Calgary, Calgary, AB, Canada; ^4^Department of Computer Science, University of Calgary, Calgary, AB, Canada

**Keywords:** machine learning, atrial fibrillation, risk prediction, random survival forest, Cox proportional-hazard models

## Abstract

**Background:**

Atrial fibrillation (AF) is a commonly encountered cardiac arrhythmia associated with morbidity and substantial healthcare costs. While patients with cardiovascular disease experience the greatest risk of new-onset AF, no risk model has been developed to predict AF occurrence in this population. We hypothesized that a patient-specific model could be delivered using cardiovascular magnetic resonance (CMR) disease phenotyping, contextual patient health information, and machine learning.

**Methods:**

Nine thousand four hundred forty-eight patients referred for CMR imaging were enrolled and followed over a 5-year period. Seven thousand, six hundred thirty-nine had no prior history of AF and were eligible to train and validate machine learning algorithms. Random survival forests (RSFs) were used to predict new-onset AF and compared to Cox proportional-hazard (CPH) models. The best performing features were identified from 115 variables sourced from three data domains: (i) CMR-based disease phenotype, (ii) patient health questionnaire, and (iii) electronic health records. We evaluated discriminative performance of optimized models using C-index and time-dependent AUC (tAUC).

**Results:**

A RSF-based model of 20 variables (CIROC-AF-20) delivered an overall C-index of 0.78 for the prediction of new-onset AF with respective tAUCs of 0.80, 0.79, and 0.78 at 1-, 2- and 3-years. This outperformed a novel CPH-based model and historic AF risk scores. At 1-year of follow-up, validation cohort patients classified as high-risk of future AF by CIROC-AF-20 went on to experience a 17.3% incidence of new-onset AF, being 24.7-fold higher risk than low risk patients.

**Conclusions:**

Using phenotypic data available at time of CMR imaging we developed and validated the first described risk model for the prediction of new-onset AF in patients with cardiovascular disease. Complementary value was provided by variables from patient-reported measures of health and the electronic health record, illustrating the value of multi-domain phenotypic data for the prediction of AF.

## Introduction

Atrial Fibrillation (AF) is the most common arrhythmia encountered in clinical practice, affecting over 30 million patients worldwide (1, 2). Beyond the age of 40, ~26% of men and 23% of women will develop AF (3, 4), a diagnosis associated with elevated risk of cardioembolic stroke (5), reduced quality of life (4), and higher risk of heart failure (HF) related events (6–9). Targeted efforts to develop and validate AF risk scores have been described (6, 8, 10–12) leveraging data from healthy populations without cardiovascular disease. The Framingham Heart Study (6), Atherosclerosis Risk in Communities (ARIC) Study (12), and Cohorts for Heart and Aging Research in Genomic Epidemiology (CHARGE)-AF consortium (8) each constructed risk models with modest predictive accuracy. The C2HEST score demonstrated superior performance through broader inclusion of patient phenotypic features (11). However, while patients with established cardiovascular disease experiencing greatest incident risk of AF (4), no risk model has been developed in this population.

The prediction of cardiac outcomes in diseased referral populations is anticipated to require a central emphasis on patient-specific disease phenotypes followed by their contextualization to patient demographics, comorbid states, current pharmacologic care, and cardiovascular symptoms. In this study we tested the predictive utility of multi-domain data resources being routinely captured at time of diagnostic testing for the prediction of time to future AF in patients with cardiovascular disease. This was tested in 7,639 consecutive patients referred to cardiovascular magnetic resonance (CMR) at two tertiary care referral institution. Collective data resources were provided to machine learning based modeling for the patient-specific prediction of time to future AF. Prediction performance using machine learning was then compared to traditional statistical modeling using a Cox proportional-hazard models and published AF risk models.

## Materials and methods

### Dataset available for risk modeling

**Figure 1:**
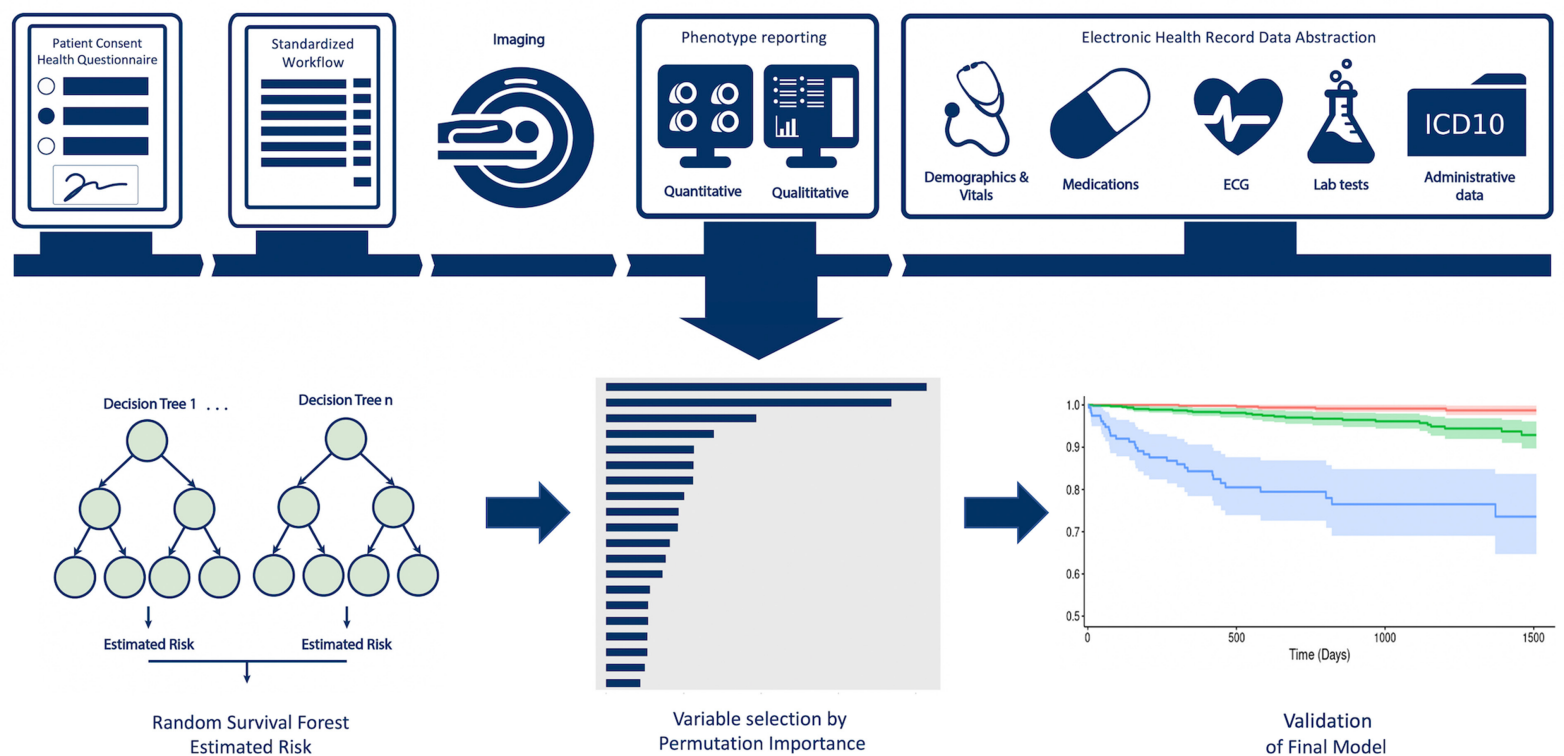
Central Illustration providing an overview of the multi-domain data collection and modeling process.

Data from 9,448 unique patients was available from the Cardiovascular Imaging Registry of Calgary (CIROC, NCT04367220), a prospective clinical outcomes study of the Libin Cardiovascular Institute. Patients referred for CMR at two tertiary care centers were engaged at time of diagnostic testing to provide informed consent and complete a standardized patient health questionnaire. All imaging studies were triaged, protocolled, and interpreted using EHR-integrated software (cardioDI^TM^, Cohesic Inc, Calgary) for the standardized collection of qualitative and quantitative phenotypic markers. Electronic health data was abstracted from the institutional data warehouse to provide patient-related laboratory, pharmacy, 12-lead ECG, Holter, and ICD-10 coded diagnostic and procedural data, as shown in Figure 1. Patients enrolled between February 2015 and November 2019 subsequently completing a minimum follow-up of 120 days were considered for model development and validation.

For the purposes of the described prediction model, all patients with a prior history of AF were excluded followed by the exclusion of patients with complex congenital heart disease (given their unique data model). Of 9,448 unique Registry patients, 7,802 met inclusion criteria with 7,639 having completed 120 days of clinical follow-up.

### Data element generation and collection

#### Patient reported health data

A standardized patient reported health (PRH) questionnaire was used to collect baseline demographic information, inclusive of ethnicity, education level, employment status, comorbid cardiac and non-cardiac diseases, alcohol consumption, smoking history, patient-reported shortness of breath based upon the New-York Heart Association (NYHA) classification, and QoL using the EQ-5D tool (13).

#### CMR imaging-based disease phenotype

CMR imaging was performed on 3-T clinical scanners (Prisma or Skyra, Siemens Healthcare, Erlangen, Germany) using standardized protocols inclusive of breath-held cine and late gadolinium enhancement (LGE) imaging in sequential short-axis views and 2-,3-, and 4-chamber long axis views. Quantitative image analyses were performed using commercial software (cvi42; Circle Cardiovascular Inc., Calgary). Left ventricular (LV) and right ventricular (RV) volumes and function were assessed on short axis cine images using semi-automated contour tracing of the endocardial and epicardial borders followed by manual adjustment. Maximal left atrial volume was assessed in the phase immediately prior to mitral valve opening using the bi-plane area-length method. All measurements were indexed to body surface area (BSA), where appropriate, using the Mosteller formula (14). Chamber volumes, mass and function were coded by z-score comparison to age and sex-based reference values (15). LGE images were scored for the presence, distribution, and burden of fibrosis, as previously described (16, 17). All other disease features were coded in accordance with guidelines provided by the SCMR and European Association of Cardiovascular Imaging (EACVI) or the American Society of Echocardiography (ASE) (18, 19).

#### Electronic health record-derived data

Electronic health information was abstracted from the institutional data warehouse, inclusive of laboratory, pharmacy, 12-lead ECG, Holter, and ICD-10 coded diagnostic and procedural data. ICD-10 coding was abstracted from the Discharge Abstract Database (DAD) and the National Ambulatory Care Reporting System (NACRS). 12-lead ECG and Holter data were obtained from archival systems (MUSE and MARS, GE Healthcare Milwaukee, USA) using custom scripts to extract vendor-coded detection of AF and identify text-based reporting of AF through internally validated natural language processing. Mortality data was obtained from Vital Statistics Alberta.

#### Primary clinical outcome

Patients were followed for the primary outcome of new-onset AF, defined as one or more of the following: (i) ICD-10 coded admission for AF (I48.0-I48.2, I48.9), atrial flutter (Aflut: I48.3-I48.4), (ii) any 12-lead ECG or Holter-based detection of AF, (iii) ICD-10 coded direct-current (DC) cardioversion (1HZ09) or ablative procedure (025S3ZZ, 025T3ZZ) for the treatment of AF. Atrial flutter was included in the primary outcome due to common co-existence, similar clinical management, and sequelae. A 2-month blackout period was applied to ensure outcomes were unrelated to any clinical events triggered by performance of diagnostic testing. The primary outcome was described in days from index CMR test performance.

### Statistical analysis

Descriptive statistics are reported as mean ± standard deviation (SD) for continuous variables with categorical variables expressed in counts with percentages. Categorical data were compared using the chi-square test/Fisher's exact test, continuous data compared using Mann-Whitney U test for non-parametric variables and independent *t*-tests for dependent variables. Missing data points were excluded from comparison for respective variables. A total of 115 variables routinely captured at time of patient encounter by the CIROC Registry were considered for risk modeling (Supplementary Table 1), inclusive of imaging-based disease phenotype (*n* = 33), patient-reported health measures (*n* = 48), and EHR abstracted variables (*n* = 34). Variables with rare missing data (< 15%) were imputed using Multivariate Imputation *via* Chained Equations (MICE) (20).

### Variable selection and model development

Population data was split into training and validation datasets using 5-fold cross validation. In this process four training folds were combined (80%) and the remaining fold (20%) reserved as a hold-out for model validation. We performed a nested cross-validation for feature selection and hyperparameter tuning. Due to the relatively rare nature of new-onset AF, each outer fold was stratified to ensure balanced event rates across folds. The validation cohorts were used for estimation of final model performance and generalizability. Missing data was imputed using Python Scikit-Learns single iterative imputer (20) separately in each fold of the cross-validation process to ensure no data leakage.

Six independent risk models were trained to predict new-onset AF over 4-years of clinical follow-up. These included two random survival forest (RSF)-based models, a novel penalized Cox proportional-hazard (CPH) model using the least absolute shrinkage and selection operator (LASSO) for variable selection, and three CPH models based on variables from published AF risk scores [C2HEST (11), Aronson et al. (10), and CHARGE-AF (8)]. For CPH models, non-linearities in continuous variables were modeled using restricted cubic splines (21) and tested to ensure proportional hazard assumptions were satisfied by way of regression analysis relating Schoenfeld residuals to time. Clinical records were reviewed for patients taking anti-arrhythmic and anti-coagulant drugs to confirm prescription for non-AF related conditions.

**Figure 2:**
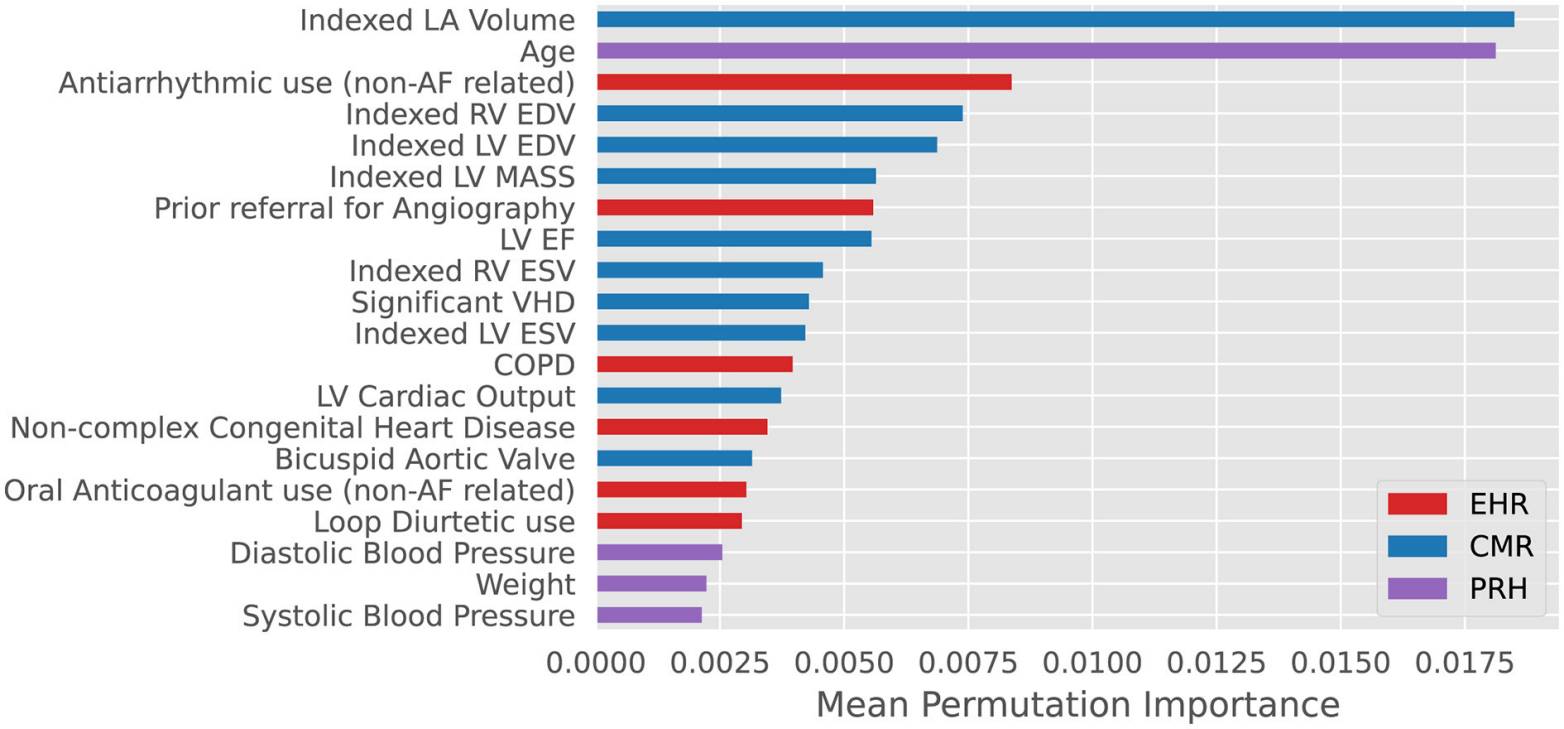
Top 20 variables for prediction of new-onset atrial fibrillation ranked by mean permutation importance calculated over 100 bootstrap samples of training data within each fold of cross-validation. VHD: valvular heart disease defined as ≥ moderate mitral or aortic valve insufficiency or stenosis. COPD: Chronic Obstructive Pulmonary Disease. EHR, Electronic Health Records; CMR, Cardiac Magnetic Resonance; PRH, Patient Reported Health (Questionnaires).

RSF-based modeling was performed to consider non-linear interactions between variables and risk contribution to future events (22), an extension of Random Forest algorithms for right censored survival data (23). RSF also are fully data driven and independent of model assumption and can handle high dimensional data without the need for apriori feature selection (24). A RSF model was selected for its capacity to deliver an explainable prioritization of contributory model features in the form of permutation importance rank, this aimed at allowing for direct comparison to variables selected by traditional statistical modeling. First, we trained a RSF using all eligible (*n* = 115) CIROC variables (CIROC-AF-115). Second, with desire for a clinically translatable model, and recognizing that removal of variables with low predictive value can improve performance (25), we constructed a parsimonious RSF model using the 20 top performing variables (CIROC-AF-20), as shown in Figure 2. Variable performance was established by calculating each variable's permutation importance over 100 bootstrap samples from within the nested training cohort and training an RSF on each bootstrapped sample for the prediction of new-onset AF. Each variable's permutation importance was determined by the out-of-bag sample for each forest and its average importance calculated across the bootstraps (Figure 2). To determine optimal hyperparameters for each RSF-based model we performed an exhaustive grid search using a nested 5-fold CV in the training cohort (Supplementary Table 2). In the same fashion, the alpha parameter for LASSO was determined by hyperparameter tuning within the nested folds. Within each training fold data for LASSO CPH modeling was normalized to zero mean and unit variance, while categorical variables were one-hot encoded.

### Performance evaluation

Each model's performance was assessed by discrimination and calibration measures. For discrimination we calculated the C-index, describing each model's ability to correctly rank event-free survival from patient scores, and the integrated brier score, which reports a measure of model performance over all time points. We reported mean C-index and integrated brier score over the five validation folds. Since C-index is shift invariant, time-dependent AUC is superior for assessing temporally sensitive risk predictions (26, 27) and was calculated at 1-, 2-, and 3-years, as well as mean value over the study duration. To assess calibration, we plotted the mean difference between predicted and observed rates of new-onset AF at each decile of risk for the best performing model's validation set, using 500 bootstrap estimates to generate 95% confidence intervals. Finally, for each risk model we compared the number needed to diagnose (NND) and the number needed to predict (NNP) at 1-, 2-, and 3-years to permit a comparison of clinical utility across models. NND estimates the number of patients who must be evaluated to correctly detect the disease of interest, NNP the number to correctly predict this disease will occur in the future (28); the former being insensitive to variation in disease prevalence. All statistical analysis and modeling were performed in Python 3.6 and R 3.6.3. Model development and validation were done in accordance with the TRIPOD reporting guidelines (Supplementary Table 3).

## Results

### Study population characteristics

The baseline characteristics of 7,639 patients contributing to each prediction model are presented in Table 1. The mean age was 52.2 ± 15.7 years with 40.8% female. The prevalence of hypertension, diabetes and coronary artery disease was 33, 12, and 11%, respectively. Referral indications are provided in Supplementary Table 4. Imaging features showed a mean left ventricular ejection fraction (LVEF) of 55.5 ± 13.7%, right ventricular ejection fraction (RVEF) of 55.1 ± 9.7%, indexed left ventricular mass (LVMi) of 59.8 ± 19.9 g/m^2^, and indexed left atrial volume (LAVi) of 35.9 ± 14.1 ml/m^2^.

**Table 1:** Baseline Clinical Demographics in patient with and without the primary outcome of incident atrial fibrillation.

**Baseline** **characteristics**	**Total cohort** **(*N* = 7,639)**	**Event –** **(*N* = 7,325)**	**Event +** **(*N* = 314)**	***p*-value**
Age (years)	52.2 ± 15.7	51.8 ± 15.7	62.1 ± 12.9	**< 0.001**
Male, *n* (%)	4,520 (59.2)	4,301 (58.9)	219 (69.7)	**< 0.001**
BSA (m^2^)	1.9 ± 0.2	1.9 ± 0.2	2.0 ± 0.3	**0.002**
BMI (kg/m^2^)	28.1 ± 6.2	28.0 ± 6.2	28.8 ± 6.7	**0.024**
SBP (mmHg)	116.4 ± 17.4	116.3 ± 17.4	117.0 ± 18.2	0.532
DBP (mmHg)	68.7 ± 12.3	68.7 ± 12.2	67.4 ± 13.1	0.076
NYHA class III or IV, *n* (%)	1,127 (14.8)	1,071 (14.6)	56 (17.8)	0.136
Previous angioplasty, *n* (%)	666 (8.7)	624 (8.5)	42 (13.4)	**0.004**
Previous bypass, *n* (%)	196 (2.6)	186 (2.5)	10 (3.2)	0.599
Smoker, *n* (%)	1,230 (16.1)	1,173 (16.0)	57 (18.2)	0.352
Alcohol consumption (>2 drinks per day), *n* (%)	203 (2.6)	197 (2.7)	6 (1.9)	0.509
Caffeine consumption (>2 drinks per day), *n* (%)	952 (12.5)	899 (12.3)	53 (16.9)	**0.020**
**Comorbidities** ^**a**^				
Diabetes, *n* (%)	928 (12.1)	867 (11.8)	61 (19.4)	**< 0.001**
Hypertension, *n* (%)	2,531 (33.1)	2,378 (32.5)	153 (48.7)	**< 0.001**
Hyperlipidemia, *n* (%)	1,225 (16.0)	1,150 (15.7)	75 (23.9)	**< 0.001**
COPD, *n* (%)	201 (2.6)	183 (2.5)	18 (5.7)	**< 0.001**
Hypothyroidism, *n* (%)	582 (7.6)	565 (7.7)	17 (5.4)	0.163
Hyperthyroidism, *n* (%)	104 (1.4)	99 (1.4)	5 (1.6)	0.911
**Medication use**				
ACE-I or ARB, *n* (%)	3,498 (45.8)	3,309 (45.2)	189 (60.2)	**< 0.001**
Antiarrhythmics^b^, *n* (%)	116 (1.5)	94 (1.3)	22 (7.0)	**< 0.001**
Anti-coagulant^b^, *n* (%)	673 (8.8)	619 (8.5)	54 (17.2)	**< 0.001**
Beta blocker, *n* (%)	3,397 (44.5)	3,202 (43.7)	195 (62.1)	**< 0.001**
Calcium channel blocker, *n* (%)	995 (13.0)	933 (12.7)	62 (19.7)	**0.006**
Digoxin, n (%)	92 (1.2)	83 (1.1)	9 (2.9)	**0.013**
Oral hypoglycemic, *n* (%)	981 (12.8)	915 (12.5)	66 (21.0)	**< 0.001**
Statin, *n* (%)	2,768 (36.2)	2,602 (35.5)	166 (52.9)	**< 0.001**
Loop diuretic, *n* (%)	855 (11.2)	782 (10.7)	73 (23.2)	**< 0.001**
Potassium sparing diuretic, *n* (%)	966 (12.6)	907 (12.4)	59 (18.8)	**0.001**
Thiazide diuretic, *n* (%)	631 (8.3)	580 (7.9)	51(16.2)	**< 0.001**

BSA, body surface area; BMI, Body mass index; SBP, systolic blood pressure; DBP, diastolic blood pressure; NYHA, New York Heart Association; COPD, chronic obstructive pulmonary disease; ACE-I, angiotensin-converting enzyme inhibitor; ARB, angiotensin II receptor blocker.

a Comorbidities were calculated from patient report health questionnaires.

b not for the treatment of atrial fibrillation. Bold values indicate *p*-values ≤ 0.05.

Following 17,697 patient-years of follow-up with median duration of 931 days (IQR 849), 314 patients (4.1%) experienced new-onset AF (crude incidence rate: 17.7 per 1,000 patient-years, with 283 diagnosed as atrial fibrillation and 31 diagnosed as atrial flutter). Patients experiencing AF showed significant differences in characteristics across all data domains (Table 1). Patients developing new-onset AF were older, more likely male, had higher rates of diabetes, hypertension, chronic obstructive pulmonary disease (COPD), hyperlipidemia, and taking more cardiovascular medications. Imaging-based phenotype revealed significantly higher LA and LV volumes, higher LV mass, lower LVEF and RVEF, and a higher prevalence of moderate-severe valvular disease (Table 2).

**Table 2:** Baseline imaging phenotypic features in patient with and without the primary outcome of incident atrial fibrillation.

**CMR-imaging variables**	**Total cohort** **(*N* = 7,639)**	**Event –** **(*N* = 7,325)**	**Event +** **(*N* = 314)**	***p*-value**
Indexed LV EDV (ml/m^2^)	87.5 ± 29.4	87.0 ± 29.0	98.0 ± 36.6	**< 0.001**
Indexed LV ESV (ml/m^2^)	41.7 ± 28.3	41.3 ± 28.0	49.8 ± 34.0	**< 0.001**
LV EF (%)	55.4 ± 13.7	55.6 ± 13.6	52.8 ± 16.0	**< 0.001**
Indexed LV Mass (g/m^2^)	59.8 ± 19.9	59.4 ± 19.7	68.1 ± 23.0	**< 0.001**
Indexed RV EDV (ml/m^2^)	82.5 ± 23.3	82.3 ± 23.0	87.5 ± 30.1	**< 0.001**
Indexed RV ESV (ml/m^2^)	38.0 ± 17.1	37.8 ± 16.8	42.2 ± 22.1	**< 0.001**
RV EF (%)	55.1 ± 9.7	55.2 ± 9.7	53.5 ± 11.0	**0.003**
Indexed LA Volume (ml/m^2^)	35.9 ± 14.1	35.5 ± 13.8	44.3 ± 18.0	**< 0.001**
Aortic stenosis^a^, *n* (%)	124 (1.6)	101 (1.4)	23 (7.3)	**< 0.001**
Aortic regurgitation^a^, *n* (%)	68 (0.9)	57 (0.8)	11 (3.5)	**< 0.001**
Mitral stenosis^a^, *n* (%)	11 (0.1)	8 (0.1)	3 (1.0)	**0.002**
Mitral regurgitation^a^, *n* (%)	140 (1.8)	121 (1.7)	19 (6.1)	**< 0.001**

LV, left ventricular; RV, right ventricular; EDV, end diastolic volume; ESV, end systolic volume; EF: ejection fraction; LA, left atrial; All indexed values are indexed to body surface area (Mosteller formula);

a ≥= moderate stenosis or insufficiency by imaging. Bold values indicate *p*-values ≤ 0.05.

For model development and validation that 7,639 patients were spilt into 5 folds for cross validation. Each fold contains 1,527–1,528 patients, with 62–63 of them developing future atrial fibrillation in the following 4 years.

### Historical cox proportional hazard AF risk model performance

**Figure 3:**
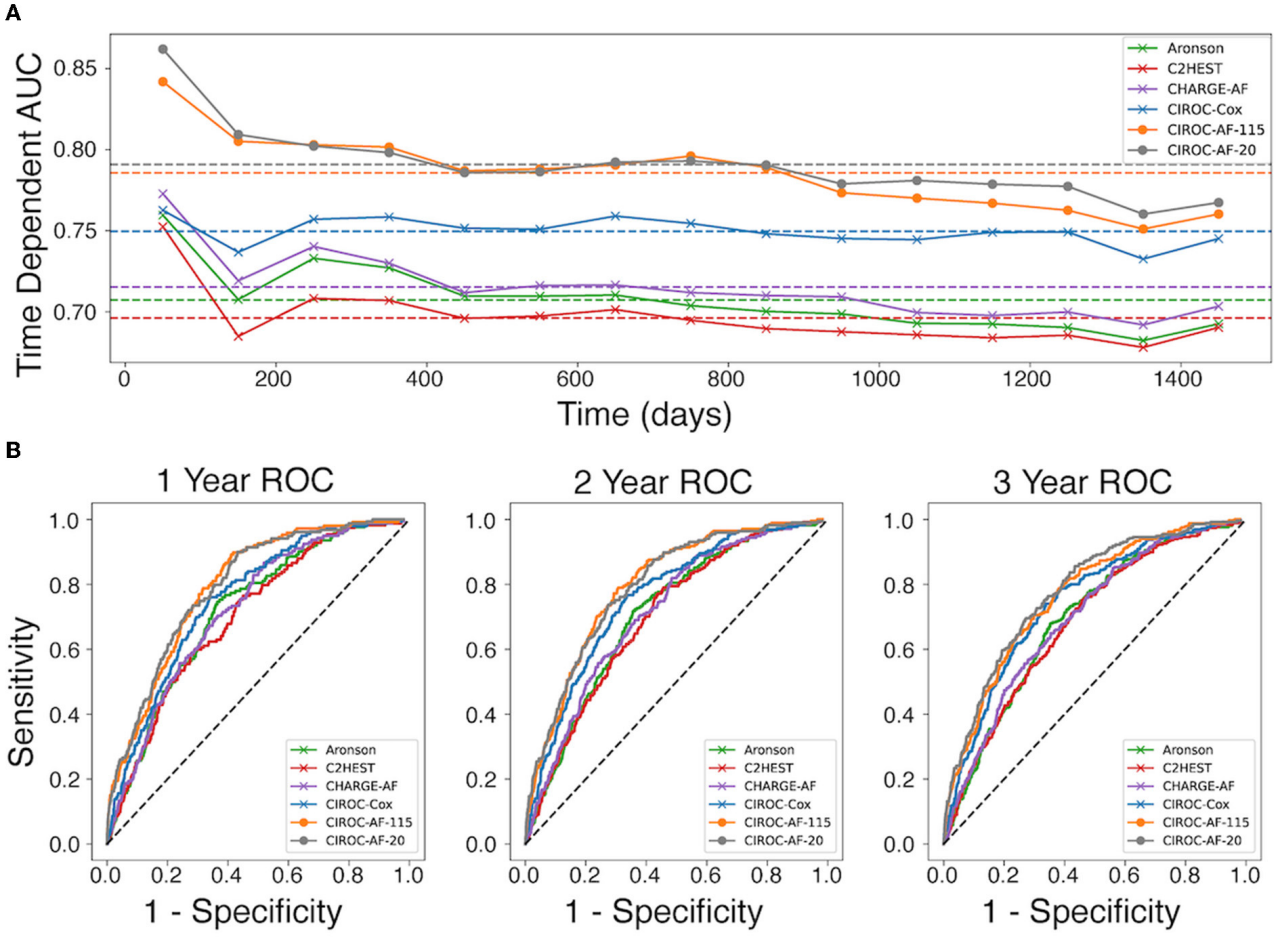
Comparison of discrimination performance for the prediction of new-onset atrial fibrillation. **(A)** Time-dependent AUC for CPH and RSF models averaged over the 5-fold validation cohorts, calculated at 15 time points for each model throughout the first 1,450 days. Dotted lines represent the mean time dependent AUC for each model. **(B)** Receiver operating characteristic (ROC) curves for each model generated at 1-year, 2-years, and 3-years.

Performance measures for CPH-based models trained using validated risk score variables (8, 10, 11) are listed in Table 3. Each showed similar discriminative performance with C-index scores of 0.70 to 0.72 averaged over the training folds. All models showed age and hypertension to be significant (*p* < 0.01) independent predictors. Model performance (mean C-index) for the C2HEST, Aronson, and CHARGE-AF models in validation datasets ranged between 0.69 and 0.71, with CHARGE-AF performing best at 0.71 ± 0.02. Each model showed a similar IBS of 0.034, indicating good performance and calibration across all time points. All models showed relatively stable validation c-indexes across each fold, with the largest difference between folds being 0.08 c-index. All historic models performed similarly over 1, 2, and 3 years by time dependent AUC (Table 4). AUC stability was modest, declining over time (Figure 3A).

**Table 3:** Historic Cox Proportional Hazard model variables and corresponding variables chosen from the CIROC Registry.

**C2HEST**	**Training mean C-index:**	**0.70** ±**0.01**	
**Original risk factors**	**CIROC variables**	**HR (95% CI)**	***p*-values**
CAD	CAD	0.93 (0.66–1.32)	0.68
COPD	COPD	1.31 (0.76–2.26)	0.33
Hypertension	Hypertension	1.43 (1.11–1.86)	**0.01**
Elderly (age>75)	Age at scan	1.04 (1.03–1.05)	**< 0.01**
Systolic HF	LVEF < 50%	0.99 (0.98–1.00)	**0.05**
Thyroid disease (hyperthyroidism)	Thyroid disease (hyperthyroidism)	0.71 (0.23–2.21)	0.86
**Aronson et al**.	**Training mean C-index:**	**0.71** ±**0.01**	
**Original risk factors**	**CIROC variables**	**HR (95% CI)**	***p*** **-values**
Age	Age at Scan	1.05 (1.04–1.06)	**< 0.01**
Female gender	Gender	0.67 (0.51–0.88)	**< 0.01**
BMI	BMI	1.02 (1.00–1.04)	0.08
SBP > 160	SBP	0.99 (0.98–1.00)	**< 0.01**
Previous MI	Previous MI	0.88 (0.63–1.22)	0.43
PAD	PAD	0.90 (0.22–3.65)	0.89
Hypertension	Hypertension	1.41 (1.08–1.84)	**0.01**
Previous HF	Previous HF	1.30 (0.88–1.92)	0.18
COPD	COPD	1.32 (0.76–2.27)	0.32
Inflammatory disease	Inflammatory disease	0.75 (0.31–1.83)	0.53
**CHARGE-AF**	**Training Mean C-index:**	**0.72** ±**0.01**	
**Original risk factors**	**CIROC variables**	**HR (95% CI)**	***p*** **-values**
Age	Age at Scan (years)	1.05 (1.04–1.06)	**< 0.01**
Race (Caucasian)	Self-Reported Ethnicity (Caucasian)	1.43 (1.05–1.95)	**0.02**
Height	Height (m)	1.57 (0.38–6.40)	0.53
Weight	Weight (kg)	1.01 (1.00–1.01)	0.13
SBP	SBP (mmHg)	0.99 (0.98–1.00)	0.08
DBP	DBP (mmHg)	0.99 (0.98–1.01)	0.33
Current smoker	Active Smoker	1.37 (0.98–1.90)	0.06
Hypertensive medication	Hypertensive Medication	1.36 (1.03–1.79)	**0.03**
Diabetes	Diabetes	1.20 (0.86–1.68)	0.29
Previous HF	Previous HF	1.26 (0.85–1.86)	0.25
Previous MI	Previous MI	0.88 (0.63–1.23)	0.46

Overall model performance in the training dataset and adjusted hazards for the primary outcome of new-onset atrial fibrillation shown. CAD, coronary artery disease; COPD, chronic obstructive pulmonary disease; HF, heart failure; LVEF, left ventricular ejection fraction; BMI, body mass index; SBP, systolic blood pressure; MI, myocardial infarction; PAD, peripheral artery disease; DBP, diastolic blood pressure. Bold values indicate *p*-values ≤ 0.05.

**Table 4:** Model discriminative performance at 1-, 2-, and 3-years, as well as overall performance by C-index and time dependent AUC.

	**Validation** **C-index** **(mean ±std)**	**Validation** **C-index** **stability** **(min–max)**	**Validation** **IBS** **(mean ±std)**	**Validation** **1-year AUC** **(mean ±std)**	**Validation** **2-year AUC** **(mean ±std)**	**Validation** **3-year AUC** **(mean ±std)**	**Validation** **Mean AUC** **(mean ±std)**
**Cox PH models**							
Aronson et al.	0.70 ± 0.03	0.67–0.75	0.034 ± 0.002	0.72 ± 0.03	0.71 ± 0.02	0.70 ± 0.02	0.71 ± 0.02
C2HEST	0.69 ± 0.02	0.65–0.73	0.034 ± 0.001	0.70 ± 0.04	0.70 ± 0.02	0.68 ± 0.02	0.70 ± 0.02
CHARGE-AF	0.71 ± 0.02	0.67–0.72	0.034 ± 0.002	0.72 ±0.03	0.71 ± 0.02	0.70 ± 0.03	0.72 ± 0.02
CIROC-AF-Cox	0.74 ± 0.02	0.71–0.79	0.033 ± 0.001	0.75 ± 0.02	0.75 ± 0.03	0.73 ± 0.03	0.75 ± 0.01
**Random** **survival forests**							
CIROC-AF-115	0.77 ± 0.02	0.74–0.79	0.031 ± 0.001	0.80 ± 0.04	0.80 ± 0.02	0.77 ± 0.02	0.79 ± 0.01
CIROC-AF-20	0.78 ± 0.01	0.75–0.81	0.031 ± 0.001	0.80 ± 0.02	0.79 ± 0.01	0.78 ± 0.02	0.77 ± 0.02

Validation: 5-fold cross-validation; C-index: Harrell's concordance index; IBS: Integrated Brier Score. AUC: time dependent (cumulative-dynamic) area under the receiver operating characteristic curve; CIROC-AF-Cox: LASSO-based variable selection. C-index Stability indicates the minimum and maximum validation c-indexes for each model across the 5-folds.

### LASSO-based cox proportional hazard model performance

The novel penalized-CPH model (CIROC-AF-Cox) reduced the variable set to 11 non-co-linear variables. CIROC-AF-Cox provided a mean C-index of 0.75 ± 0.01 over the training folds and mean validation C-index of 0.74 ± 0.02 and mean validation IBS of 0.034 ± 0.001. It showed similarly stable validation across each of the 5-folds (Table 4). CIROC-AF-Cox showed time-dependent AUC values at 1-, 2- and 3-years of 0.75 ± 0.02, 0.75 ± 0.03, 0.73 ± 0.03, and 0.75 ± 0.01, respectively. CIROC-AF-Cox showed improved stability in AUC values over time vs. historic models (Figure 3A).

### Machine learning based AF risk prediction model performance

Our novel RSF-based models showed improved discrimination performance vs. historic CPH-based models, and vs. our novel CIROC-AF-Cox model. The CIROC-AF-115 model achieved a mean C-index of 0.77 ± 0.02, with the parsimonious CIROC-AF-20 model providing similar performance with mean C-index of 0.78 ± 0.01. Both RSF models had mean IBS of 0.033 ± 0.001 and showed model stability on par with the best CPH model (CHARGE-AF) with a maximum variation of 0.05 c-index between the folds. RSF models also outperformed CPH based approaches when assessed by time-dependent AUC. CIROC-AF-115 provided respective AUCs at 1-, 2- and 3-years of 0.80, 0.80, and 0.77 while CIROC-AF-20 provided respective AUCs of 0.80, 0.79, and 0.78 (Table 4).RSF Model stability was similar to the CPH models, declining slightly over the 4 year study time (50 days−1,450 days) (Figure 3A).

**Figure 4:**
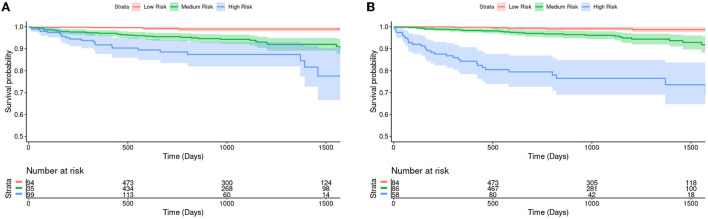
Kaplan-Meier survival curves and hazard ratios for risk of new-onset atrial fibrillation based on tertiles of predicted risk by **(A)** CIROC-AF-Cox and **(B)** CIROC-AF-20 models. The shaded area indicates a 95% confidence interval. Number at risk indicates the number of patients each model has predicted to be within each group at a given time. Intermediate risk is an estimated risk of > 1.5% and < 4%, where high risk is patients estimated at a risk of > 4%. These curves show a single fold's model performance on the fold's validation set. The log rank test *p*-values between each survival curve are shown in the table and have been adjusted *via* the Benjamini-Hochberg Procedure.

CIROC-AF-20 and CIROC-AF-Cox models were compared to determine how they correctly predicted low, intermediate, and high risk of incident AF. High risk was considered a predicted risk >4% per year, chosen as a 10-fold higher rate than the general population (29). Low risk was considered < 1.5%. As shown in Figure 4, predicted risk estimates appropriately discriminated the future occurrence of AF. High risk patients predicted by CIROC-AF-20 experienced a 24.7-fold higher rate of AF at 1-year, 14.3-fold at 2-years, and 13.0-fold at 3-years vs. low-risk patients (*p* < 0.001 for all).

### Time interval-based AUC performance and calibration

**Figure 5:**
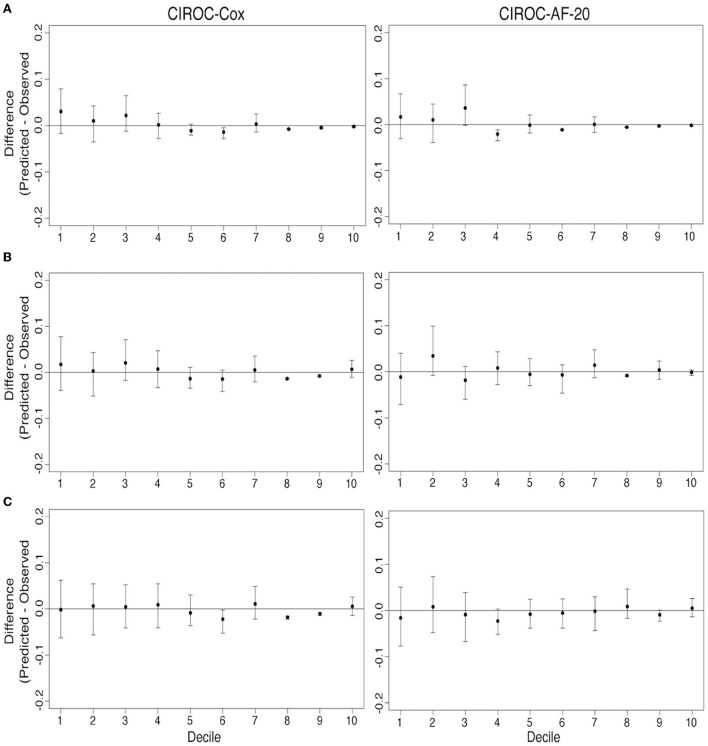
Comparison of model calibration for CIROC-AF-Cox and CIROC-AF-20 for new-onset atrial fibrillation prediction at **(A)** 1-year, **(B)** 2-years, and **(C)** 3-years. Differences between predicted and observed event rates is plotted across each decile of predicted risk. Black points indicate estimates from validation data sets and error bars indicate the 95% confidence interval from 500 bootstrapped validation data sets.

AUC curves for each model generated at 1-, 2-, and 3-years are shown in Figure 3B. RSF-based models showed improved discrimination across all time intervals vs. CIROC-AF-Cox and historic risk models. Calibration plots describing observed vs. predicted probabilities of new-onset AF at a 1-, 2-, and 3-years are shown in Figure 5. Both novel models showed good calibration across all deciles of predicted risk.

### Clinical diagnostic performance

To compare diagnostically relevant performance markers, NND and NNP were calculated at 1-, 2- and 3-years. RSF models consistently outperformed CIROC-AF-Cox and all historic CPH models. RSF based models showed lowest NND between 1.97 and 2.32, with NNP ranging from 4.73 to 15.73 (Table 5).

**Table 5:** Number needed to diagnose (NND) and number needed to predict (NNP) performance indicators for all constructed prediction models of new-onset atrial fibrillation.

	**1-Year NND (mean ±std)**	**2-Year** **NND** **(mean ±std)**	**3-Year NND (mean ±std)**	**1 Year** **NNP** **(mean ±std)**	**2 Year NNP (mean ±std)**	**3 Year** **NNP** **(mean ±std)**
**Cox PH models**						
Aronson et al.	2.57 ± 0.66	2.74 ± 0.65	3.01 ± 0.64	17.91 ± 2.33	11.00 ± 2.67	6.58 ± 1.12
C2HEST	2.82 ± 0.47	2.92 ± 0.43	3.22 ± 0.46	21.29 ± 2.42	12.25 ± 2.30	7.02 ± 0.61
CHARGE-AF	2.51 ± 0.32	2.61 ± 0.26	2.96 ± 0.41	21.57 ± 4.56	10.53 ± 1.45	6.57 ± 1.27
CIROC-AF-Cox	2.17 ± 0.19	2.24 ± 0.32	2.36 ± 0.29	16.56 ± 4.32	8.71 ± 1.86	5.08 ± 0.70
**Random survival** **forests**						
CIROC-AF-115	1.97 ± 0.16	1.99 ± 0.08	2.32 ± 0.19	15.52 ± 1.04	8.00 ± 0.36	5.19 ± 0.53
CIROC-AF-20	2.03 ± 0.13	2.04 ± 0.09	2.18 ± 0.14	15.73 ± 1.77	7.62 ± 0.75	4.73 ± 0.57

NND: number needed to diagnose; the number of patients who need to be examined in order to correctly detect one person with the disease of interest in a study population of persons with and without the known disease. NNP: Number needed to predict; the number of patients who need to be examined in the patient population in order to correctly predict the diagnosis of one person.

## Discussion

This study demonstrated the capacity for machine learning to deliver accurate patient-specific predictions of future AF occurrence in patients with cardiovascular disease using routinely reported CMR phenotypic markers contextualized to patient-reported and EHR-abstracted health information. Versus historic AF risk models (8, 10, 11) we observed significant performance gains through expanded access to multiple data domains and through the use of machine learning-based methods.

With the exception of two studies focused in critically ill patients (30, 31), machine learning-based predictions of incident AF have been restricted to community practice settings using administrative health record data (32, 33). Despite the limited translation of these models to cardiovascular disease referral populations, these studies provided foundational evidence for machine learning to provide incremental value for the prediction of incident AF. Hill et al., used administrative health data from the UK Clinical Practice Research Datalink (CPRD) to predict future AF occurrence from 18 variables, delivering an AUC of 0.827 at 10-years vs. 0.725 using the CHARGE-AF risk score (32). A subsequent study confirmed similar findings but highlighted that much of the observed value in this referral population was being provided by conventional AF risk factors (33). Due to a low annual incidence of AF in community population settings, both studies required long term surveillance (e.g., 10-years) to identify patients at a meaningful risk of incident AF, this significantly limiting future implementation of cost-effective surveillance strategies. The alternate consideration of diagnostic testing data to assist in machine learning-based AF prediction has, to date, focused on 12-lead ECG data (34). In a single study, a model trained from ECG vector data in a community referral population showed potential for the identification of patients at elevated risk. However, whether such approaches can discriminate risk in patients with cardiovascular disease (where ECG abnormalities are more consistently observed) remains unknown. Our study uniquely focused on the prediction of AF risk in patients undergoing diagnostic imaging for cardiovascular disease, demonstrating the complementary value of disease phenotypic markers, patient-reported health measures, and EHR-abstracted health information to inform risk modeling. Importantly, all these data assets were routinely captured by, or automatically migrated to a central reporting solution. By eliminating any need for manual data collection or abstraction at time of diagnostic testing this study offers pragmatic evidence for the real-world delivery of multi-domain data collection in routine clinical practice.

As shown in Table 3, many predictors adopted by historical AF risk models (in primary care populations) failed to reach significance in patients with cardiovascular disease. Our machine learning based model objectively chose seven of the top 10 predictive variables from the imaging-based phenotype data domain. LA volume ranked first, a marker recognized as a dominant predictor of AF in both healthy (35–38) and disease-specific cohorts (29, 39). Left atrioventricular coupling index (LACI) and its change have also been shown to have an independent association with new-onset AF in the Multi-Ethnic Study of Atherosclerosis (MESA) (40). Incrementally, LVEF, LVEDVi and LV mass were important contributors; the latter acknowledged by CHARGE-AF (8). Of interest, RV EDVi was highly ranked, justifying value for multi-chamber phenotyping using CMR.

The cumulative risk of new-onset AF in our cardiovascular disease population was 4.1% at a median follow-up of 2.6 years; representing 17.7 AF events per 1,000 patient-years. This event confirms a higher incident risk of AF in this referral population vs. primary care where incident rates are between 4.0 and 6.7 events per 1,000 person-years (6, 8, 12, 29). This unique risk distribution emphasizes the need for population-specific risk models.

Finally, new-onset AF represents an ideal disease target for personalized prediction modeling at time of diagnostic testing given the availability of validated therapies for reduction of cardio-embolic risk (41). With our model's observed 17.8% 1-year incident rate of new-onset AF in patients classified to be high-risk, actionable justification exists for the implementation of surveillance programs using Holter or wearable device-based tools for the prevention of AF-related cardiovascular events.

### Limitations

Several important limitations are recognized for the current study. Our study was performed at two tertiary care hospitals within the same healthcare system. The initial study only validated the model through cross-validation and needs further hold-out validation and accordingly, external validation prior to model implementation beyond our local institution. Incremental model calibrations through expanded population exposures are also advisable for all risk models, particularly to address varying ethnic distributions (42). Of the 7,639 studied patients, 5,195 (68%) were Caucasian. At time of risk modeling, the CIROC Registry had prospectively tracked clinical outcomes for a period of 4 years, and therefore uncertainty remains in the capacity of the presented model to deliver risk estimation beyond this period. Our models were trained using CMR-specific phenotypic variables. Matched echocardiographic data was not routinely available given high rates of private outpatient laboratory use, as is commonly encountered in cardiology practice. Accordingly, direct comparison to similar models trained from echocardiographic variables was not feasible. Implementation for other imaging modalities requires unique variable training and validation, recognizing unique differences in variable characteristics and referral bias. Similarly, our study did not include patients with congenital heart disease given unique anatomic phenotypes and disease profiles to routine adult cardiovascular disease. Accordingly, the current risk model is not applicable to this patient population. Finally, alternate machine learning-based techniques can be exploited for the prediction of outcomes from complex health data (32) and are planned for future investigation. In this inaugural study we did not comprehensively examine the comparative performance of alternate machine learning methodologies for survival-based prediction. Future research aimed at optimizing the presented AF prediction tool using alternate models is planned.

## Conclusions

In this study we demonstrated capacity for multi-domain patient data collected at time of CMR-based phenotyping to support machine learning-based prediction of future AF in patients with cardiovascular disease. As the first described prediction model of AF risk in a cardiovascular disease population, our optimized model identified de-novo patients who experienced a 25-fold higher risk of incident AF over a 12-month period. This work provides foundational support for phenotype-based prediction modeling at time of diagnostic imaging for the delivery of personalized care. Future studies assessing the impact of AF prediction modeling at time of diagnostic imaging are warranted.

## Data availability statement

The raw data supporting the conclusions of this article will be made available by the authors, without undue reservation.

## Ethics statement

The studies involving human participants were reviewed and approved by Conjoint Health Research Ethics Board at the University of Calgary. The patients/participants provided their written informed consent to participate in this study.

## Author contributions

SD performed all data analysis, statistical, machine learning based modeling, and manuscript authorship. JW was senior author and conceived, designed, edited, and finalized manuscript content. All other authors either assisted in data collection and adjudication (DL, YM, LL, AC, JF, SR, RS, AS, and PF), study design and manuscript review (NF, CL, AH, CM, SW, and DE) or data modeling (MG). All authors contributed to the article and approved the submitted version.

## Funding

This study was partially funded by the Calgary Health Foundation.

## Conflict of interest

Authors JW, AH, and JF each contributed to development of the novel software platform that is now supported by Cohesic Inc., and hold equity (shares) in this company. Author JW is the Chief Medical Officer of Cohesic Inc. Author JW has received research funding from Siemens Healthineers, Circle Cardiovascular Inc., and Pfizer Inc. Author AH has received funding from Amgen. Author SD receives funding from Alberta Innovates. The remaining authors declare that the research was conducted in the absence of any commercial or financial relationships that could be construed as a potential conflict of interest.

## Publisher's note

All claims expressed in this article are solely those of the authors and do not necessarily represent those of their affiliated organizations, or those of the publisher, the editors and the reviewers. Any product that may be evaluated in this article, or claim that may be made by its manufacturer, is not guaranteed or endorsed by the publisher.

## Abbreviations

AF: Atrial Fibrillation
AUC: Area Under the Receiver Operator Characteristic Curve
CIROC: Cardiovascular Imaging Registry of Calgary
CPH: Cox Proportional-Hazards
CV: Cross Validation
EHR: Electronic Health Record
NND: Number Needed to Diagnose
NNP: Number Needed to Predict
RSF: Random Survival Forest
SCD: Sudden Cardiac Death
VHD: Valvular Heart Disease.
