# Supplementary material for: Machine learning prediction of atrial fibrillation in cardiovascular patients using cardiac magnetic resonance and electronic health information
Source: Front Cardiovasc Med. 2022 Sep 28;9:998558. doi: 10.3389/fcvm.2022.998558 (PMC9554748; doi:10.3389/fcvm.2022.998558)
Supplement: Supplementary file 1 [file Data_Sheet_1.docx]

**Supplemental Tables**

**Supplemental Table 1.** Overview of the variables available for risk modelling.

| **Modelling Variables** | | | | | |
| --- | --- | --- | --- | --- | --- |
| **Demographics** | **Known Cardiovascular Conditions** | **Known Non- Cardiovascular Conditions** | **Medications** | **Imaging Phenotypic Markers** | **Family History** |
| Age | Aorta disease | Amyloidosis | ACE Inhibitors | Indexed left ventricular end diastolic volume | Coronary artery disease |
| Sex | Arrhythmogenic right ventricular cardiomyopathy | Asthma | ARBs | Indexed left ventricular end systolic volume | Myocardial infarction |
| Ethnicity | Bicuspid aortic valve | Cancer | Antiarrhythmics | Left ventricular ejection fraction | Arrhythmogenic right ventricular cardiomyopathy |
| Height | Cerebrovascular disease | Chronic obstructive pulmonary disease | Anti-Coagulants | Left ventricular end diastolic volume | Sudden cardiac death |
| Weight | Congenital heart disease | Fabry's disease | Beta Blockers | Left ventricular end systolic volume | **Previous Medical Encounter** |
| Body Surface Area | Coronary artery disease | Hemochromatosis | Calcium Channel Blockers | Indexed left ventricular mass | Previous hospitalization |
| Body Mass Index | Dilated cardiomyopathy | Inflammatory bowl disease | Digoxin | Left ventricular mass | Previous myocardial infarction |
| Systolic Blood Pressure | Diabetes | Kidney disease | Oral Hypoglycemics | Left ventricular cardiac output | Previous angiogram |
| Diastolic Blood Pressure | Heart failure | Liver disease | Statins | Indexed right ventricular end diastolic volume | Previous angioplasty |
| Quality of Life (EuroQOL) | Valve disease | Marfan's syndrome / Ehlers’s Danlos syndrome | Loop Diuretics | Indexed right ventricular end systolic volume | Previous bypass |
| Ethnicity | Hypertension | Multiple myeloma | Potassium Sparing Diuretics | Right ventricular ejection fraction |  |
| Marital status | Hyperlipidemia | Muscular dystrophy | Thiazide Diuretics | Right ventricular end diastolic volume |  |
| Education level | Hypertrophic cardiomyopathy | Rheumatoid arthritis | Insulin | Right ventricular end systolic volume |  |
| Employment status | Peripheral arterial disease | Sarcoidosis | Aspirin | Indexed left atrial volume |  |
| Smoker | Pulmonary hypertension | Sickle cell anemia | Ivabradine | Aortic regurgitation |  |
| Alcohol Consumption | Ventricular Tachycardia | Systemic Lupus erythematosus | Entrestro | Aortic stenosis |  |
| Caffeine Consumption |  | Thalassemia |  | Mitral Regurgitation |  |
| Soda Consumption |  | Hypothyroidism |  | Mitral stenosis |  |
| NYHA Class |  | Hyperthyroidism |  | Valvular Heart Disease |  |
| CCS |  |  |  | Late Gadolinium Enhancement - Fibrosis Patterns |  |
|  |  |  |  |  |  |
| NYHA: New York Heart Association; CCS: The Canadian Cardiovascular Society Angina Grade; ACE: Angiotensin-converting enzyme; ARBs: Angiotensin receptor blockers; | | | | | |

**Supplemental Table 2.** Breakdown of patients by their referral indication.

| **Referral Indication** | **Total Cohort (N=7639)** | **Event -**  **(N=7325)** | **Event +**  **(N=314)** |
| --- | --- | --- | --- |
| Aortic Disease | 163 | 144 | 19 |
| Cardiac Mass | 123 | 116 | 7 |
| Cardio-Oncology | 575 | 563 | 12 |
| Congenital | 503 | 483 | 20 |
| Coronary Artery Disease | 1217 | 1154 | 63 |
| Electrophysiology | 401 | 382 | 19 |
| Iron Overload | 86 | 83 | 3 |
| Myocarditis | 697 | 681 | 16 |
| Non-Ischemic Cardiomyopathy | 3238 | 3120 | 118 |
| Dilated Cardiomyopathy | 1206 | 1169 | 47 |
| Hypertrophic Cardiomyopathy | 611 | 581 | 30 |
| Amyloid | 177 | 167 | 10 |
| Sarcoid | 232 | 227 | 5 |
| Other | 1012 | 985 | 24 |
| Pericardial Disease | 93 | 92 | 1 |
| Poor Echo | 4 | 4 | 0 |

**Supplemental Table 3**. TRIPOD Checklist: Prediction Model Development and Validation.

| **Section/Topic** | **Item** |  | **Checklist Item** | **Page** |
| --- | --- | --- | --- | --- |
| **Title and abstract** | | | | |
| Title | 1 | D;V | Identify the study as developing and/or validating a multivariable prediction model, the target population, and the outcome to be predicted. | 1 |
| Abstract | 2 | D;V | Provide a summary of objectives, study design, setting, participants, sample size, predictors, outcome, statistical analysis, results, and conclusions. | 1 |
| **Introduction** | | | | |
| Background and objectives | 3a | D;V | Explain the medical context (including whether diagnostic or prognostic) and rationale for developing or validating the multivariable prediction model, including references to existing models. | 2 |
|  | 3b | D;V | Specify the objectives, including whether the study describes the development or validation of the model or both. | 2 |
| **Methods** | | | | |
| Source of data | 4a | D;V | Describe the study design or source of data (e.g., randomized trial, cohort, or registry data), separately for the development and validation data sets, if applicable. | 2-3 |
|  | 4b | D;V | Specify the key study dates, including start of accrual; end of accrual; and, if applicable, end of follow-up. | 2 |
| Participants | 5a | D;V | Specify key elements of the study setting (e.g., primary care, secondary care, general population) including number and location of centres. | 2 |
|  | 5b | D;V | Describe eligibility criteria for participants. | 3 |
|  | 5c | D;V | Give details of treatments received, if relevant. | N/A |
| Outcome | 6a | D;V | Clearly define the outcome that is predicted by the prediction model, including how and when assessed. | 3 |
|  | 6b | D;V | Report any actions to blind assessment of the outcome to be predicted. | N/A |
| Predictors | 7a | D;V | Clearly define all predictors used in developing or validating the multivariable prediction model, including how and when they were measured. | Fig 1 / Tbl 3 /  Sup 4 |
|  | 7b | D;V | Report any actions to blind assessment of predictors for the outcome and other predictors. | 4 |
| Sample size | 8 | D;V | Explain how the study size was arrived at. | 3 |
| Missing data | 9 | D;V | Describe how missing data were handled (e.g., complete-case analysis, single imputation, multiple imputation) with details of any imputation method. | 4 |
| Statistical analysis methods | 10a | D | Describe how predictors were handled in the analyses. | 4 |
|  | 10b | D | Specify type of model, all model-building procedures (including any predictor selection), and method for internal validation. | 4 |
|  | 10c | V | For validation, describe how the predictions were calculated. | 5 |
|  | 10d | D;V | Specify all measures used to assess model performance and, if relevant, to compare multiple models. | 5 |
|  | 10e | V | Describe any model updating (e.g., recalibration) arising from the validation, if done. | N/A |
| Risk groups | 11 | D;V | Provide details on how risk groups were created, if done. |  |
| Development vs. validation | 12 | V | For validation, identify any differences from the development data in setting, eligibility criteria, outcome, and predictors. | 6 |
| **Results** | | | | |
| Participants | 13a | D;V | Describe the flow of participants through the study, including the number of participants with and without the outcome and, if applicable, a summary of the follow-up time. A diagram may be helpful. | 3 |
|  | 13b | D;V | Describe the characteristics of the participants (basic demographics, clinical features, available predictors), including the number of participants with missing data for predictors and outcome. | Tbl 1 / Sup 4 |
|  | 13c | V | For validation, show a comparison with the development data of the distribution of important variables (demographics, predictors and outcome). | N/A |
| Model development | 14a | D | Specify the number of participants and outcome events in each analysis. | 5 |
|  | 14b | D | If done, report the unadjusted association between each candidate predictor and outcome. | N/A |
| Model specification | 15a | D | Present the full prediction model to allow predictions for individuals (i.e., all regression coefficients, and model intercept or baseline survival at a given time point). | CPH – Tbl 3  RSF – Fig 1 / Sup 3 |
|  | 15b | D | Explain how to the use the prediction model. | 3 |
| Model performance | 16 | D;V | Report performance measures (with CIs) for the prediction model. | 6 |
| Model-updating | 17 | V | If done, report the results from any model updating (i.e., model specification, model performance). | 6 |
| **Discussion** | | | | |
| Limitations | 18 | D;V | Discuss any limitations of the study (such as nonrepresentative sample, few events per predictor, missing data). | 8 |
| Interpretation | 19a | V | For validation, discuss the results with reference to performance in the development data, and any other validation data. | N/A |
|  | 19b | D;V | Give an overall interpretation of the results, considering objectives, limitations, results from similar studies, and other relevant evidence. | 8 |
| Implications | 20 | D;V | Discuss the potential clinical use of the model and implications for future research. | 8 |
| **Other information** | | | | |
| Supplementary information | 21 | D;V | Provide information about the availability of supplementary resources, such as study protocol, Web calculator, and data sets. | 8 |
| Funding | 22 | D;V | Give the source of funding and the role of the funders for the present study. | 9 |

**Supplemental Table 4.** Nested hyperparameter grid search,

| **Variable** | **Tested Range** | **Selected Value** |
| --- | --- | --- |
| Max Depth | 3, 5, 10, 20, 40, None | **20** |
| Max Features | sqrt(N), log(N) | **sqrt(N)** |
| Minimum Cases in Leaf | 1, 2, 5, 10, 15 | **10** |
| Minimum Samples for Split | 2, 5, 10 | **5** |
| Number of Trees | 10, 50, 100, 200, 400 | **200** |
